# Identification of an error in the implementation of the Eleveld model in a commercial TCI pump

**DOI:** 10.1007/s10877-025-01333-8

**Published:** 2025-07-29

**Authors:** Nicolas Milliet

**Affiliations:** Jules Gonin Eye Hospital, 15, avenue de France, CP 1, Lausanne, 1001 Switzerland

I have identified a significant discrepancy in the implementation of the Eleveld general-purpose model for propofol in a commercial target-controlled infusion (TCI) pump. As an anaesthetist with experience in TCI—including the Marsh and Schnider models—I have closely followed the development of the Eleveld model. This model is notable for its applicability across a wide range of patient populations, including adults, children, the elderly, and obese patients. It is designed to automatically adjust for patient-specific parameters such as age, sex, height, and actual body weight. Compared to older models, the Eleveld model is particularly advantageous for obese patients. While it recognizes that heavier patients require higher doses to achieve a defined plasma or effect-site concentration, it uses mathematical formulas to allometrically scale clearance and adjust compartment sizes. This ensures that the increase in dose with obesity is not linear but tailored to individual patient characteristics.

## Case description

In 2024, our institution adopted the B. Braun Space + infusion pumps, which implement the Eleveld model for propofol. Our initial experience was very positive, with the model proving user-friendly once its principles were understood. However, during a recent case involving a 63-year-old male patient (173 cm, 100 kg, BMI > 30), I observed that the induction bolus calculated by the pump (162 mg at a Ce target of 4 mcg/ml using the Eleveld + opioids mode) appeared abnormally low. A simulation using TivaTrainer, employing the same parameters, resulted in a substantially higher calculated bolus dose (192 mg). This discrepancy prompted further investigation.

## Investigation and Findings

Suspecting a potential implementation error, I contacted the manufacturer’s representative and provided detailed case information. Simultaneously, I consulted with Dr. Frank Engbers, developer of the TivaTrainer simulation tool. Together with Dr. Hernán Boveri, we systematically compared the pump’s output with simulation results across a range of patient profiles.

- A mix-up between the two Eleveld model modes (with and without opioids) was quickly excluded.
- The hypothesis that the error stemmed from the formula calculating a unique k_eO_ for each patient was also ruled out.

**Fig. 1:**
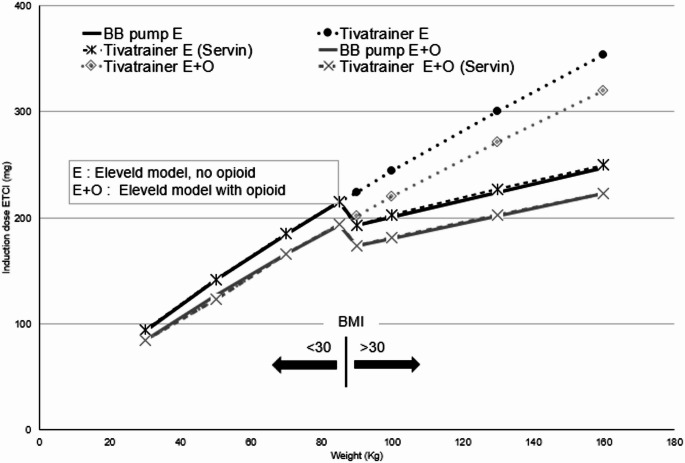
Induction bolus versus total body weight in a theoretical 40-year-old male (170 cm) targeting an effect-site concentration of 4 mcg/ml using both Eleveld model modes (with and without opioids)

We then compared the induction dose delivered by the pump to the simulated induction bolus in a theoretical patient of increasing weight (see Table 1). While the bolus delivered by the pump was comparable to the one simulated with TivaTrainer in non-obese patients (BMI < 30), we found that the induction bolus delivered by the pump became much lower as soon as the theoretical patient met the criteria for obesity (BMI > 30). This cutoff corresponded to the point at which the induction bolus curve showed an abrupt, unexpected downward shift (see Fig. 1).

**Table 1:**
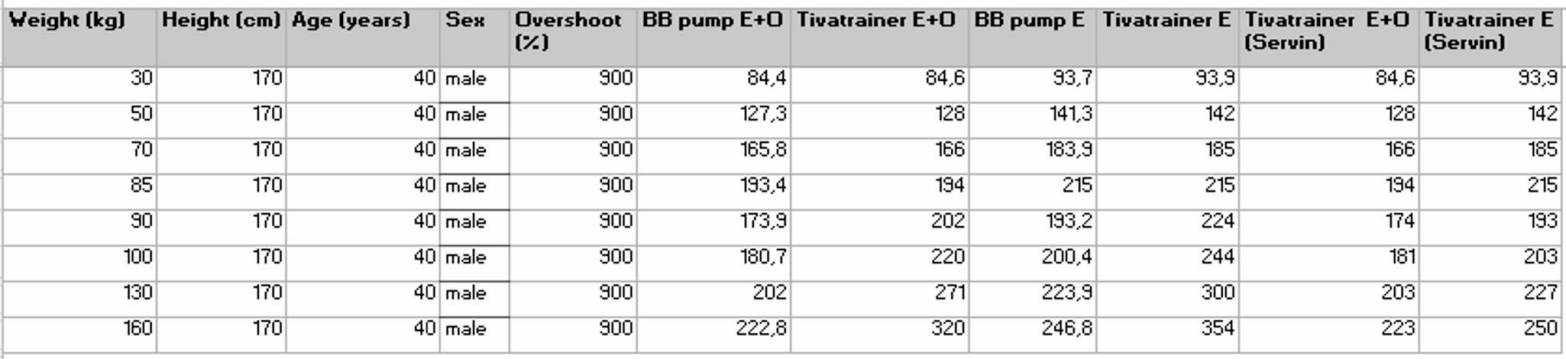
Induction bolus doses (mg) required to achieve an effect site concentration of 4 mcg/ml using both modes of the Eleveld model (with and without opioids) in a theoretical 40-Year-Old male (170 cm) of varying weights

BB Pump E + O: B.Braun Space + delivery using the Eleveld Model with opioid; BB Pump E: B.Braun Space + delivery using the Eleveld Model without opioid; Tivatrainer E + O: Tivatrainer simulation with the Eleveld Model with opioid; Tivatrainer E: Tivatrainer Simulation using the Eleveld Model without opioid; Tivatrainer E + O (Servin): Tivatrainer simulation using the Eleveld Model with opioid with modified weight according to Servin’s formula when BMI is > 30; Tivatrainer E (Servin): Tivatrainer simulation using the Eleveld Model without opioid with modified weight according to Servin’s formula when BMI is > 30.

Note the marked decrease in pump-delivered dose when BMI exceeds 30; BB Pump E + O: B.Braun Space + delivery using the Eleveld Model with opioid; BB Pump E: B.Braun Space + delivery using the Eleveld Model without opioid; Tivatrainer E + O: Tivatrainer simulation with the Eleveld Model with opioid; Tivatrainer E: Tivatrainer Simulation using the Eleveld Model without opioid; Tivatrainer E + O (Servin): Tivatrainer simulation using the Eleveld Model with opioid with modified weight according to Servin’s formula when BMI is > 30; Tivatrainer E (Servin): Tivatrainer simulation using the Eleveld Model without opioid with modified weight according to Servin’s formula when BMI is > 30; BMI: Body Mass Index (Kg /m2).

## Further Analysis

Further analysis suggested that the error might stem from an incorrect adjustment of body weight within the pump’s algorithm. Specifically, it appeared that the pump’s software applied Servin’s formula (“Input Weight = Total Body weight – 0.4 x [Total Body weight – Ideal Body Weight]) to change the weight of obese patients (BMI > 30), rather than using actual (total) weight for all patients as required by the Eleveld model. This was later confirmed after the manufacturer’s technical team consulted with Dr. Douglas Eleveld, the model’s developer.

TivaTrainer simulation data using both Eleveld model modes (with and without opioids)—with Total Body Weight for BMI < 30 and with modified weight according to Servin’s formula for BMI > 30—are also provided in Table 1. This approach results in simulations that match the actual induction bolus delivered by the pump well (see Table 1; Fig. 1). Notably, as the degree of obesity increases, the reduction in the induction dose delivered by the pump becomes more pronounced, meaning that higher levels of obesity are associated with a greater percentage decrease in the administered dose—an effect consistent with Servin’s formula (see Table 1).

We used Devine’s formula to estimate Ideal Body Weight to calculate the “Input Weight “according to Servin’s formula. We believe this choice explains the slight discrepancy between the bolus induction dose delivered by the pump and the results obtained by simulation, especially as our theoretical patient becomes very obese. Using a different formula to estimate IBW would have yielded slightly different results.

The manufacturer has acknowledged the error and plans to release a software update to correct this issue.

## Discussion

This case underscores several important points:

1. **Value of Simulation Tools**: Tools such as TivaTrainer are invaluable for verifying the theoretical drug delivery of TCI models against actual pump performance, enabling early detection of implementation errors.
2. **Need for Rigorous Certification**: Infusion pumps are medical devices whose embedded pharmacokinetic/pharmacodynamic algorithms must be rigorously validated and certified to ensure correct implementation before market release.
3. **Importance of Reporting Platforms**: Establishing a centralized platform for clinicians to report and discuss potential pump malfunctions or discrepancies could facilitate faster identification and resolution of such issues.

## Conclusion

This case highlights the critical need for robust validation and post-market surveillance of TCI pump software, especially as models become more sophisticated and are applied to diverse patient populations. I hope this report will encourage further vigilance and the development of systematic approaches for monitoring and improving the safety of infusion devices.

## Data Availability

No datasets were generated or analysed during the current study.
